# Ultrasound generation in water via quasi-periodically snapping polymeric core–shell micro-bead excited with radiowaves

**DOI:** 10.1038/s41598-024-56614-0

**Published:** 2024-03-12

**Authors:** Salvatore Buonocore, Aliaksandr Hubarevich, Francesco De Angelis

**Affiliations:** Istituto Italiano Di Tecnologia, Via Morego 30, 16163 Genova, Italy

**Keywords:** Optics and photonics, Physics

## Abstract

This work reports the results of a theoretical and numerical study showing the occurrence of stochastically resonating bistable dynamic in polymeric micro-bead of sub-micrometric size with stiff core and soft shell. The system, submerged in water, is excited with a pulsed laser working in the Mega-Hertz frequency range and tuned to match both an optical and acoustic resonance of the system. The laser interacts with the carbon nanotubes embedded in the shell of the polymeric micro-bead generating heat. The concurrent action of the generated heat with the standing acoustic oscillations, gives rise to a stochastically resonating bistable system. The system in fact is forced to switch between two states (identifiable with the creation and organized disruption of a quasi-hexagonal tessellation) via a snap-through-buckling mechanism. This phenomenon results in the unprecedented generation of pressure oscillations. These results open the way to develop a new type of core–shell micro-transducers for radioacoustic imaging applications able to work in the Mega-Hertz frequency range. From a more general thermodynamic perspective, the reported mechanism shows a remarkable periodicity and energy conversion efficiency.

## Introduction

Deep tissue penetration of light is still a key scientific challenge both in basic and applied science with a clear breakthrough in diagnostic and therapeutic applications. Corresponding technical implementations would be transformative for the healthcare sector with strong impacts on the society. In particular, the possibility of real time and non-invasive total body imaging represents a revolutionary diagnostic method. In fact, current approaches such as X-Ray CT and NMR present evident drawbacks such as high costs, complex and demanding instrumentations and, in case of X-Ray serious side effects. This situation arises from the fact that there are just few electromagnetic spectral windows in which the human body is transparent enough to let radiation penetrate deep into the tissue while producing low or no side effects. Among these few spectral windows, the radio waves band represents an unexplored possibility. In fact, radio waves can penetrate deeply into biological tissues with no side effects due to their extremely low energy. A possible approach to exploit radio waves is the use of a nano-transducer able to optically absorbing low energy electromagnetic waves. The consequent heat generation and local mechanical expansion will bring to ultrasounds emission that can be used for local imaging. Such a radio-acoustic imaging would enable total body diagnostics with spatial resolution reaching few microns and with no side effects. Importantly, as to be used in-vivo, the size of such a transducer must be in the micro- or nano-scale. Thus, the size of the transducer is orders of magnitude smaller than the exciting radio wavelength ($\lambda$ = 3.3 m @12 MHz in water). Until now, this extreme condition looked far to be reached. However, the tremendous advances achieved in nano-optics and metamaterials opened unprecedented possibilities that make this challenge no longer prohibitive. In a previous work, we showed the possibility of creating a new class of micro-transducers enabling total body thermo-acoustic imaging in the radio range. To this aim, we introduced two new concepts that act in a synergic manner: Radio-Plasmonics and Resonant Thermoacoustic Expansion^1^. Radio-Plasmonics consists in the possibility of exciting surface plasmons at megahertz frequency to promote resonant optical absorption and then enhanced heat generation. In fact, as showed by different groups, by doping polymers or dielectric matrices with carbon based or metallic nanomaterials is possible to achieve a new metamaterial that behaves in the radio-frequency range in the same way noble metals behaves in the visible/NIR range (conventional Plasmonics). In analogy with conventional plasmonics, such micro-transducers will exhibit a more effective light to heat conversion and then a stronger ultrasound emission. Furthermore, in the radio-range the transducers can be designed to show acoustic resonances that provide acoustic oscillations of higher amplitude and long-lasting duration. We called this approach resonant thermo-acoustic expansion. In other words, the underlying idea is to mix electromagnetic resonances (resonant absorption in radio-plasmonics) with acoustic resonances (resonant expansion) to achieve super-resonant light-to-sound conversion in the radio range for imaging purposes. In this work, the boundary of this synergetic combination is moved forward by designing acoustic micro-transducers characterized by bistable states excited by stochastic resonances. Such a bistable dynamic allows to further increase the intensity of the generated ultrasounds and then to decrease the particle size from the micro- to the nano-range. To push the limit of particle size we investigated the behavior of the system at 385 MHz, where the subwavelength condition (particle size vs light wavelength) is less stringent. This frequency is not far from the spectral band legally reserved for medical implants in humans (MICS band 401–406 MHz). In this regime, we achieved ultrasound pressures in the order of 1–10 Pascal for particles of 250 nm in radius, thus comparable to results achievable with gold nanoparticles in the NIR range. These numbers suggest the plausibility of a new generation of ultrasound imaging techniques based on bistable micro transducers and plasmonic metamaterials in the radio range. In this respect, in light of the theory introduced in^1^, we foresee three options to modulate the occurrence of resonant optical absorption in the MICS band: (1) Modulate the radius (and therefore the volume) of the micro-bead. (2) Modify the permittivity of the CNT doping material by adjusting their type and/or percentage weight. (3) Exploit the variation with temperature and salinity of the environment permittivity ${\epsilon }_{h}$ in order to obtain the sought plasmonic resonance in the targeted region of the human body. For what concerns the acoustic spectra of the micro-bead they can be calculated by the acoustic scattering of a solid (PDMS) sphere submerged in water when perturbed by a planar acoustic wavefield. The procedure suggests that the acoustic resonances of the spectra can be easily tuned to match specific spectral windows. In fact, let us recall that changing the Young’s modulus of the material E changes its speed of sound and consequently the acoustic resonance of the particle as ${f}_{acou resonance}\sim \frac{{c}_{s}}{d}\sim \frac{\sqrt{\frac{E}{\rho }}}{d}$. In light of this last relation, we foresee two options to modulate the occurrence of acoustic resonances in a selected frequency band: (1) Modulate the radius (and therefore the volume) of the micro-bead and (2) modulate the elastic modulus of its constituents and their distributions (inertia moments). However, as recalled in^1^, the effectiveness of the proposed working mechanism relies on the concept of a *doubly resonant system*. In this respect, to effectively excite the acoustic resonance it is necessary a perturbation frequency close to its value i.e. ${{f}_{exc}\sim f}_{acou resonance} .$ In case of single pulse excitation, this relation imply a pulse lasting approximately half-acoustic cycle. That is, the excitation of an acoustic resonance at a given frequency is maximized by an excitation pulse of a given duration FWHM. Therefore, given that for quasi-harmonic pulse shape holds the relation ${f}_{exc}\sim \frac{1}{2\cdot FWHM}$, a maximized response is obtained when $\frac{1}{2\cdot FWHM} \sim {c}_{s}$. Therefore, an optimal response is achieved when the frequency of the excitation pulse matches both the plasmonic resonance and the acoustic resonance. The double matching is key for maximizing the optical absorption and the heat generation on a time scale allowing the excitation of an acoustic resonance.

For sake of clarity, let us recall the concepts of bistability and stochastic resonance for non-technical readers. The expert readers can directly move to the next paragraph.

Broadly speaking, a bistable system has an output that can assume only one of two distinct stable values indifferently from the type of input applied. The switching between these values is caused by a temporary change of the level of the input that can span across two different threshold levels, let us call them threshold "1" and "2"^2^. When the input is lower than threshold 1 the output (the system) is in the "low state"; when the input is higher than threshold 2 the system is in the "high" state. For values of the input between the two thresholds, the output keeps its value. The dual threshold action is denoted hysteresis, implying a dependence of the output of the system on the history of the input. This means the system has a “flip-flop” behavior, namely if the output is low a large positive (negative) input spike flips it to high (low). These devices, when properly biased, can have large differential gain and remarkable amplifying power. For making a bistable system are necessary two features: *nonlinearity* and *feedback*.

A remarkable example of bistable systems is provided by the snap buckling mechanisms. This mechanism is largely exploited in nature, for example by the Venus flytraps^3^ or by the pistol-shrimps^4^. It has been also revealed in multi-layered polymeric spheres and more generally in spherical shells^5^. They arise from geometric or material properties and result in a rapid transition between two stable states^6^. For example, some flexible structures, in some post buckling deformation^7^ can exhibit snap buckling when the applied load passes the critical buckling load, such as an arched shell with a central vertical load^8^. The dynamical characteristics of the collapse from one stable state to the other makes these mechanisms exploitable for storing and releasing energy for fast actuation purposes^8^.

Other renowned examples of bistable systems are the ring laser in optics and the Schmitt trigger in electronics. Notably, in both the examples has been observed the phenomenon of stochastic resonance^9,10^. This concept was initially proposed to explain the periodic reoccurrence of ice ages^11^, is nowadays used in a wide variety of systems and is interdisciplinary to many research areas (e.g. physics, biology, medicine, geology)^12,13^. Briefly, the stochastic resonance is a phenomenon in which a weak sinusoidal signal can resonate with a white noise so that the frequencies of the signal are amplified thus increasing the signal to noise ratio. When occurring in a bistable system, the stochastic resonance may boost the input signal switching from low to high and vice versa. The latter increases the dynamic behavior of the system that now can switch between the two states even in presence of a very weak variation of the input. In other words, in presence of a stochastic resonance, the bistable system usually switches in a much shorter time and in more abrupt way. The occurrence of this effect^14^ requires three main components: (1) a weak sinusoidal signal, (2) noise source and (3) an energetic activation barrier.

**Figure 1:**
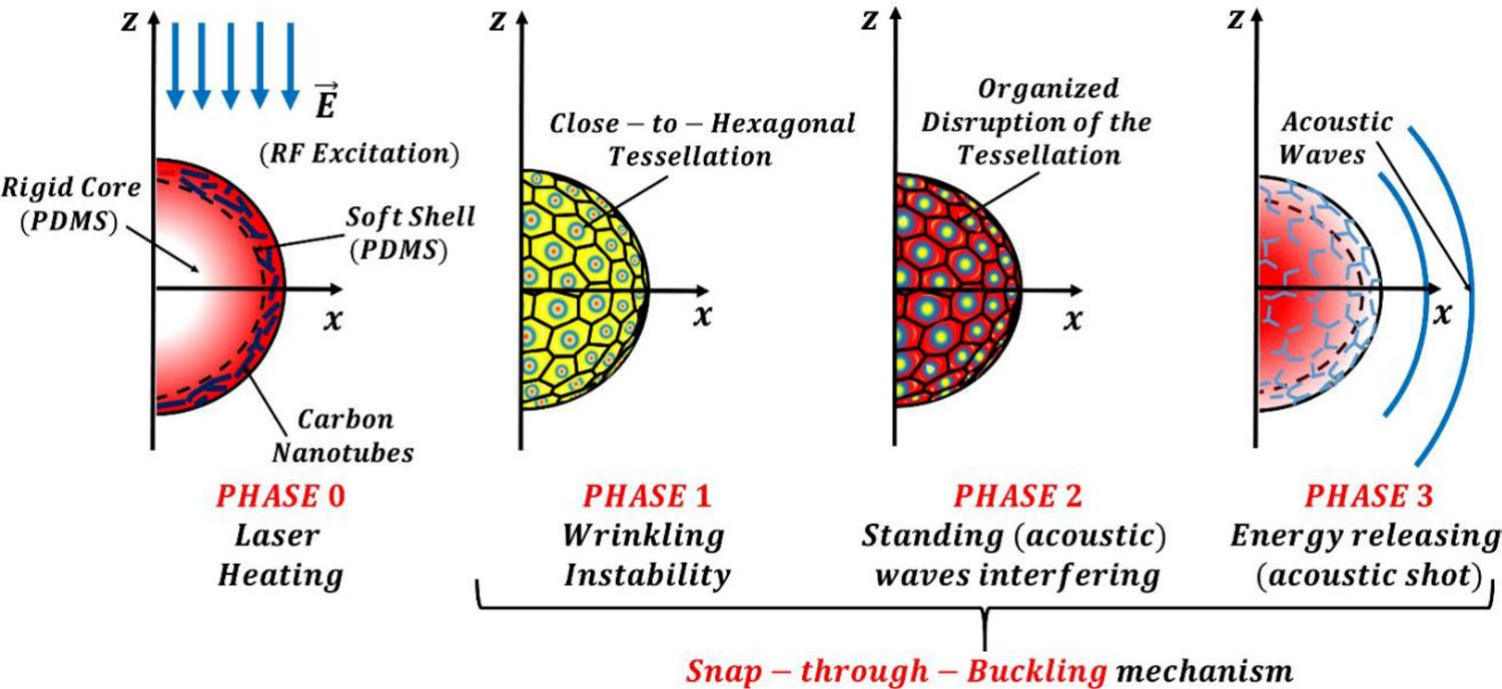
Schematic of the four phases characterizing the stochastic bistable resonance in the polymeric micro-bead. *Phase 0*: Single Pulse Laser Heating. *Phase 1*: Quasi-hexagonal tessellation (via wrinkling instability). *Phase 2*: Organized disruption of the tessellation (via standing waves interference). *Phase 3*: Acoustic shot (via strain energy release).

In the present study, the micro transducer is composed of a core–shell polymeric micro-bead in which the core is an insulator whereas the shell is doped with carbon nanotubes (CNT) so that it is made conductive and plasmonic in the radio range. The concentration of CNT is adjusted to maximize the optical absorption and then heat generation at the desired frequency of 385 MHz. As detailed thereafter, the properties of the polymeric core–shell micro-bead are tuned to make it acoustically resonant in the same range of frequencies. As consequence, the heat generated by the radio wave pulse in the bead also activates an acoustic resonant mode. In this system, the thermal gradient represents the white noise whereas the resonant expansion and the corresponding acoustic standing waves are the weak sinusoidal signal. Therefore, the system is capable of showing a stochastic resonance. Importantly, such a core–shell system may also exhibit wrinkling instability. The latter is characterized by two dynamic states. In the "high state”, the *strain energy* of the shell is uniformly distributed within the shell itself. In the "low state" the *strain energy* is distributed in self-organized (quasi-)hexagonal cells whose size is determined by the geometric and elastic properties of the medium^15,16^. Now let us describe how the stochastic resonance can lead to the switching of the bistable system. The dynamic of the system can be represented in four phases as described below and schematically illustrated in Fig. 1.

1. Phase 0: Heating of the bead via single pulse radio waves which characteristics are set to match both an optical and acoustic resonant mode in the MHz frequency range.
2. Phase 1: Building of the inner thermal gradient (that is the noise source) that sets the conditions for the occurrence of a wrinkling instability (i.e. the formation of the quasi-hexagonal tessellation). This phenomenon constitutes the “low state” of the bistable system.
3. Phase 2: Interfering of thermal noise and the standing waves (that is the small periodic force) associated with the acoustic resonant mode. The resulting stochastic resonance amplifies the acoustic standing waves and provides the flipping spike required to reach the ”high state” of the bistable system , i.e. the organized disruption of quasi-hexagonal tessellation.
4. Phase 3: Releasing of the strain energy of the buckled cells, collected by the standing waves, in the form of a focused acoustic shot, so to recover the status to undergo Phase 1 thus preparing the system for the next cycle.

It is worth noticing that the succession of the Phases 1, 2 and 3, corresponds overall to a “snap-through-buckling” mechanism. Several groups have already investigated the (conditions of) occurrence of this mechanism in a broad plethora of structural elements theoretically and experimentally^17–19^.

## Numerical modeling of the photoacoustic phenomenon

### Electromagnetic wave propagation

The system analyzed in this study is composed of a polymeric (PDMS) core–shell micro-bead (of total diameter 1 um) that embeds carbon nanotubes in its shell (of total thickness 100 nm). A single pulse lasting 1.5 ns excites the bead. For the selected combination of geometrical characteristics and forcing light wavelength, the system has an extremely small electrical size and, though conductive, the skin depth is much larger too: in such a situation, propagation does not play a practical role and neither (auto-) induction. Therefore, the electric field distribution $\overrightarrow{E}$ mimiking the light pulse (the selected operating frequency is 385 MHz where the polymeric core–shell micro-bead embedding CNT has an optical resonance) has been computed using the Gauss law (Electric Current module in COMSOL Multiphysics). To generate the electric field, a capacitor was created by setting an appropriate potential and ground on two planar faces as described by the following equations:

1 $$\overrightarrow{J}=\sigma \overrightarrow{E}+j\omega \overrightarrow{D}, \overrightarrow{E}=-\nabla V, \overrightarrow{D}={\epsilon }_{0}{\epsilon }_{r}\overrightarrow{E},$$

where $\overrightarrow{E}$ is the electric field, $\overrightarrow{J}$ is the current density, $V$ is the electric potential, $\sigma$ is the electrical conductivity and ${\epsilon }_{0},{\epsilon }_{r}$ are the vacuum and relative permittivity, respectively. In particular ${\epsilon }_{r}$ is the (frequency dependent) complex permittivity, which value for the soft shell embedding carbon nanotubes at 12 $\mathrm{\%}$, has been extrapolated by the experimental measurements reported in literature^20^. The rigid core has been designed to be an electrical insulator. The absorbed energy can be consequently calculated as resistive heating ${Q}_{r}$ (power per unit volume):

2 $${Q}_{r}=\overrightarrow{J}\cdot \overrightarrow{E}.$$

These losses are used for thermal coupling. Let us briefly recall how the *absorption cross section* of the micro-bead is calculated in order to identify the optical resonant spectral position at 385 MHz. In the frequency range investigated, given the condition $R\ll {\lambda }_{opt}$ (where R is the particle radius and ${\lambda }_{opt}$ is the wavelength of the incident light) the particle is represented by its dipole moment $\overrightarrow{p}={\epsilon }_{0}\alpha \overrightarrow{E}$. On the other hand, the polarizability of the sphere is defined as $\alpha = {\left({\alpha }_{0}^{-1}-i\frac{{k}^{3}}{6\pi }\right)}^{-1},\mathrm{ where }{\alpha }_{0}=3 \left(\frac{4\pi {R}^{3}}{3}\right)\frac{{\epsilon }_{r}-{\epsilon }_{h}}{{\epsilon }_{r}+2 {\epsilon }_{h}}$ is the quasi-static polarizability, $k=\frac{{n}_{h}\omega }{c}$ is the wavenumber of the surrounding medium and ${n}_{h}$ is its refractive index. Now, in order to obtain a Surface Plasmon resonance, the materials’ permittivity must obey the condition ${\epsilon }_{r}\approx -2 {\epsilon }_{h}$ at the sought spectral position^1,21^. This causes a resonant behavior for the particle’s polarizability and therefore a resonant absorption in the corresponding spectrum that corresponds to the excitation of the dipolar SP of the particle. Therefore, the absorption cross section of the particle can be calculated as the absorption by a dipole moment ${\sigma }_{abs}= k(Im \left\{\alpha \right\}-\frac{{k}^{3}}{6\pi }{\left|\alpha \right|}^{2})$. The validity of the results obtained with the quasi-static approximation can be easily verified with Mie theory for spherical particles.

### Heat transfer

The temperature field distribution in the micro-bead is obtained by the diffusion equation^22^:

3 $$\rho c\frac{\partial T}{\partial t}=k{\nabla }^{2}T+{Q}_{r}*f({\tau }_{w})$$

where $\rho ,c,k$ are the density, heat capacity and thermal conductivity, respectively. In particular, the thermal conductivity is assumed constant within the material. The function $f({\tau }_{w})$ is the temporal shape of the incident pulse with width ${\tau }_{w}$ defined as the full width at half maximum (FWHM) of the Gaussian profile^22^:

4 $$f({\tau }_{w})=\frac{1}{b\sqrt{2\pi }}{e}^{-(t-{t}_{0}{)}^{2}/2{b}^{2}},$$

where ${t}_{0}$ is the time position of the center of the peak and $b$ is the standard deviation

5 $$b={\tau }_{w}/2(2ln2{)}^\frac{1}{2}.$$

### Structural mechanics

The stress and strain induced in the micro-bead material by the heat diffusion is calculated with a structural mechanics model of linear thermal expansion using the Duhamel-Hookes law:

6 $$s=C:\left(\epsilon -\alpha \left(T-{T}_{0}\right)\right) ,$$

where $s$ and $\epsilon$ indicate the total stress and strain tensors, $\alpha$ is the thermal expansion tensor, $T$ and ${T}_{0}$ the temperature variable and the reference temperature, and $C$ the fourth-order elasticity tensor. Finally, the operator $:$ indicate a double contraction. According to the model presented in^22^, the pressure generated in the aquatic environment surrounding the micro-bead by photoacoustic effect is calculated considering water as a solid domain. The stress tensor $s$ is proportional to the second time derivative of the structural displacement

7 $$\nabla s=\rho \frac{{\partial }^{2}u}{\partial {t}^{2}} .$$

The boundary conditions to be imposed at the interface of the micro-bead and the water environment is the equality between the normal acceleration for the acoustic pressure ${p}_{t}$, and the acceleration based on the second temporal derivative of structural displacement

8 $$\left(\frac{1}{\rho }\nabla {p}_{t}\right)=\frac{{\partial }^{2}u}{\partial {t}^{2}} .$$

### Acoustic wave propagation

The pressure waves generated by the photoacoustic effect induced by the micro-bead thermal expansion obeys the acoustic wave equation:

9 $$\frac{1}{\rho {c}_{s}^{2}}\frac{{\partial }^{2}{p}_{t}}{\partial {t}^{2}}-\nabla \frac{1}{\rho }\left(\nabla {p}_{t}\right)=0,$$

where ${c}_{s}$ is the speed of sound and ${p}_{t}=p+{p}_{b}$ is the total acoustic pressure as the sum of the sound pressure $p$ and ambient pressure ${p}_{b}$.

### Physical parameters

In this section are summarized the physical parameters of the proposed system. The polymeric micro-bead is composed of a hard PDMS core (Young’s modulus 30,000 kPa) and soft PDMS shell (Young’s modulus 300 kPa). The thermal properties of the core and shell region are identical. The thermal conductivity and heat capacity for PDMS are 0.15 W m^−1^ K^−1^ and 1460 J kg^−1^ K^−1^ respectively, while those for the surrounding water are 0.58 W m^−1^ K^−1^ and 4200 J kg^−1^ K^−1^.

The soft shell is doped with carbon nanotubes at 12 $\mathbf{\%}$ in weight (using the experimental values reported in literature^20^) and this corresponds to a complex refractive index value (at the forcing frequency of the laser) of (27.51–j * 30). On the other hand, the rigid core is an electric insulator, with complex refractive index value of (2–j * 0.0025). The refractive index of water at the selected forcing frequency of the laser (385 MHz) is (8.9–j * 0.01) ^23^. The system is illuminated with a single pulse laser at the radio frequency of 385 MHz, with fluence 40 J m^−2^ and a total pulse duration of 1.5 ns. For this selected parameters’ combination, specifically when the CNT are embedded in the shell of the bead, the system has a fundamental optical resonance at 385 MHz.

Overall, as largely discussed in the introduction, these parameters have been calibrated in the aim of exploiting coupled optical-thermo-acoustic resonances in the polymeric core–shell micro-bead. In fact, a simple parametric study using plane acoustic waves impinging on the polymeric micro-bead and sweeping the radio-frequency range shows the occurrence for the selected system of an acoustic fundamental resonance at 385 MHz too. In particular, the optical resonance (that is activated when the shell is doped with CNT) is responsible of the activation of a thermodynamic mechanism (i.e. heat generation and storage) which characteristics are favorable to establishing highly efficient thermoacoustic resonant dynamic. This effect is not observed in the absence of such optical resonance, as shown later on. At this frequency, the amplitude of the pressure signals generated by photoacoustic effect is maximized by a laser pulse duration of 1.5 ns^1^. For these optimized parameters, the (acoustic) resonant dynamic of the polymeric micro-bead couples sinergetically with the generated heat (via a snap-through-buckling mechanism) forming a stochastically resonating bistable system. The result is an efficient energy transduction system to the external environment as discussed in detail in the following sections. Interestingly this working mechanism occurs also when the micro-bead core is made of fluid, namely water. The reason lies in the fact that the acoustic characteristic of the selected fluid core (e.g. the acoustic wave speed) are similar to the hard-PDMS analyzed in this study. This suggests that, in line of principle, the device could work also with a gas core, therefore several photoacoustic modalities^24–28^ could potentially benefit from the proposed approach.

## Dynamic of the bistable mechanism

In this section are discussed the characteristics of the bistable dynamic observed in the physical system under investigation. Two cases will be analyzed and compared: (1) CNT are embedded solely in the shell of the polymeric particle (the core behaves as an electric insulator) and (2) CNT are in the sole core of the polymeric particle (the shell is an insulator). Therefore, in each case, the heating element corresponds with the shell region and the core region, respectively. These two cases are compared in the aim of analyzing the effect of the heat generation and storing mechanism on the resulting thermo-acoustic oscillations. It is worth to recall that only in the first case (CNT in the bead’s shell) the system is at an optical resonance. The results discussed in the following section correspond to a polymeric particle of total diameter 1 um and shell thickness 100 nm. Therefore the total volume of the core region and of the shell region are approximately equivalent.

### Phase 0: Plasmonic heating

During the first 1.5 ns, an electromagnetic pulse impinges on the polymeric particle and, interacting with the carbon nanotubes embedded in it, generates heat by Joule effect. The characteristics of the pulse are tuned to match both the plasmonic and fundamental acoustic mode of the particles therefore activating a resonant dynamic. The double resonant mechanism was discussed in a previous paper^1^ and not further reported here for brevity.

**Figure 2:**
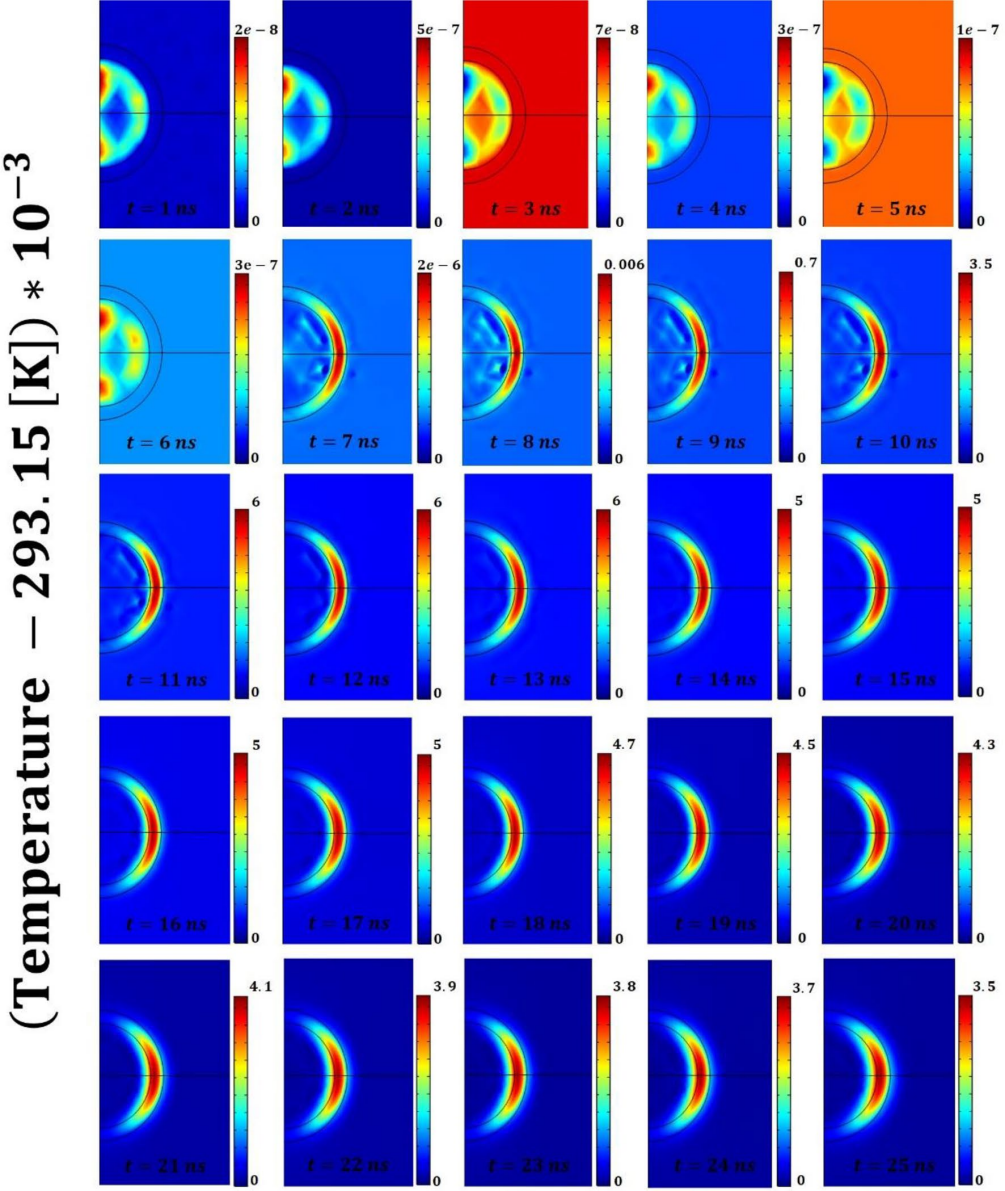
Temperature field (represented as the difference with respect to the temperature of the surrounding water 293.15[K]) within the polymeric (PDMS) core–shell micro-bead and the surrounding water environment for the initial 25 ns of the process (the laser pulse irradiation occurs during the initial 1.5 ns). Remarkable is the heat diffusion dynamic that, initiated by the heating of the shell, is mainly directed toward the micro-bead’s core. This heat storage in the core behaves as the noise-source of the stochastically resonating bistable mechanism. The colorbars have units of milli-Kelvin.

**Figure 3:**
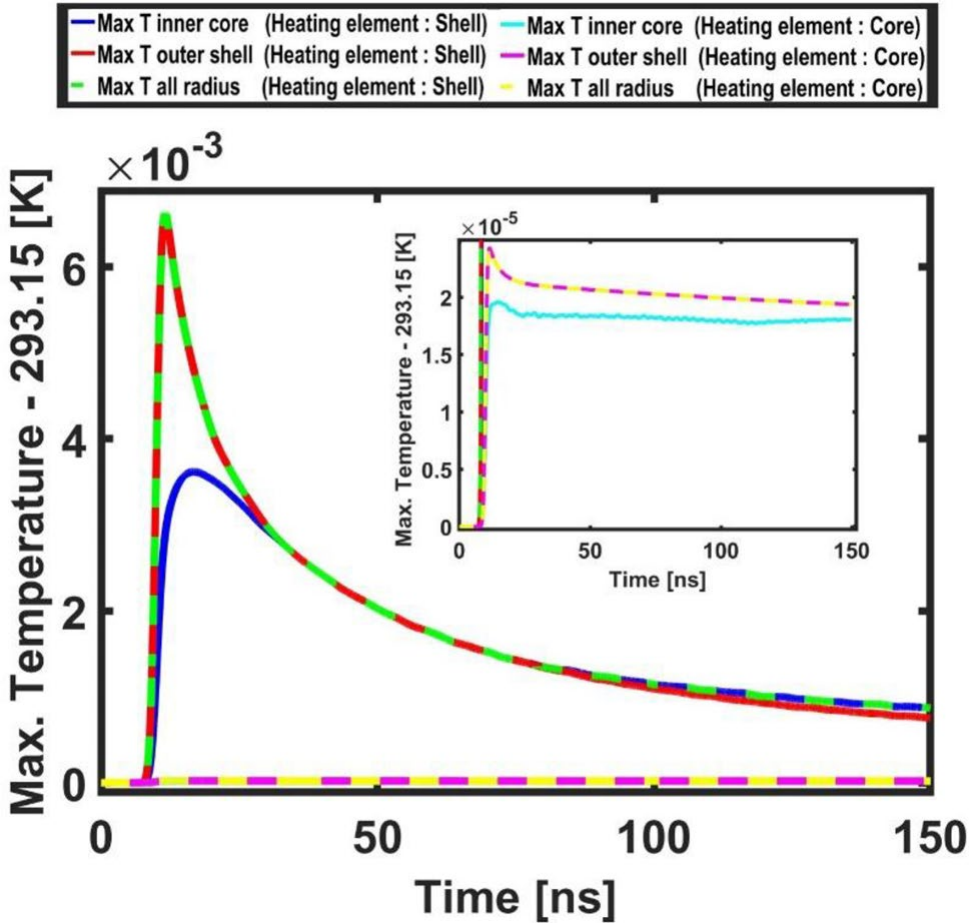
Temporal evolution of the maximum temperature values (reported as the difference with respect to the temperature of the surrounding water 293.15[K]) in the elements of the system, i.e. the core, the shell and the overall bead. Blue (ciano), red (purple) and green (yellow) lines refer respectively to the maximum temperature values on the inner core radius, outer shell radius and overall radius when the heating element is the shell (core).

**Figure 4:**
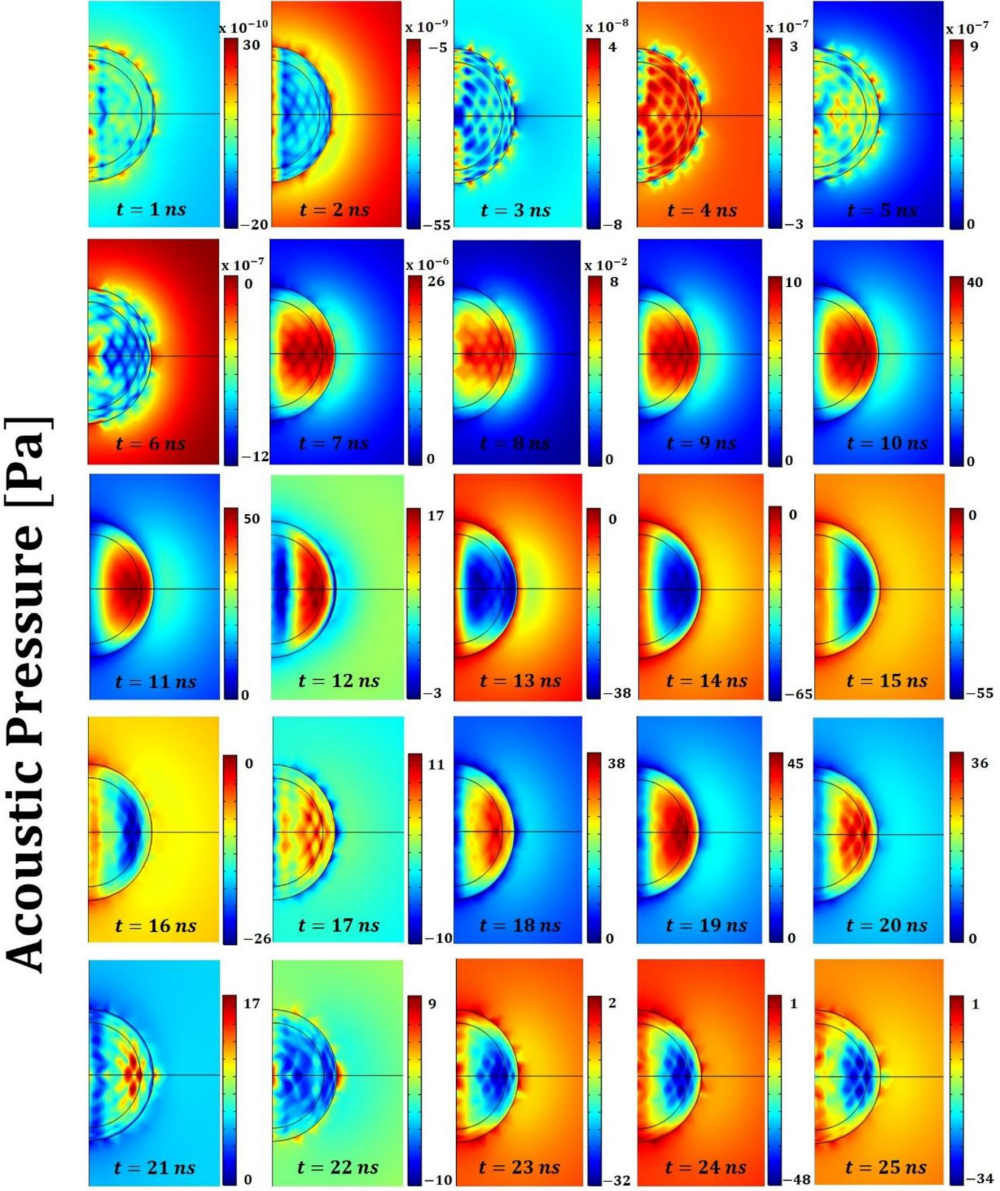
Acoustic pressure field within the polymeric (PDMS) core–shell micro-bead and the surrounding water environment for the initial 25 ns of the process. Remarkable is the occurrence of a “close-to-hexagonal” tessellation pattern that, once disrupted by (acoustic) standing waves, leads to acoustic shots (e.g. at nanosecond 22). Overall, these last mentioned phenomena constitute a snap-through-buckling mechanism. The color bars have units of Pascal [Pa].

**Figure 5:**
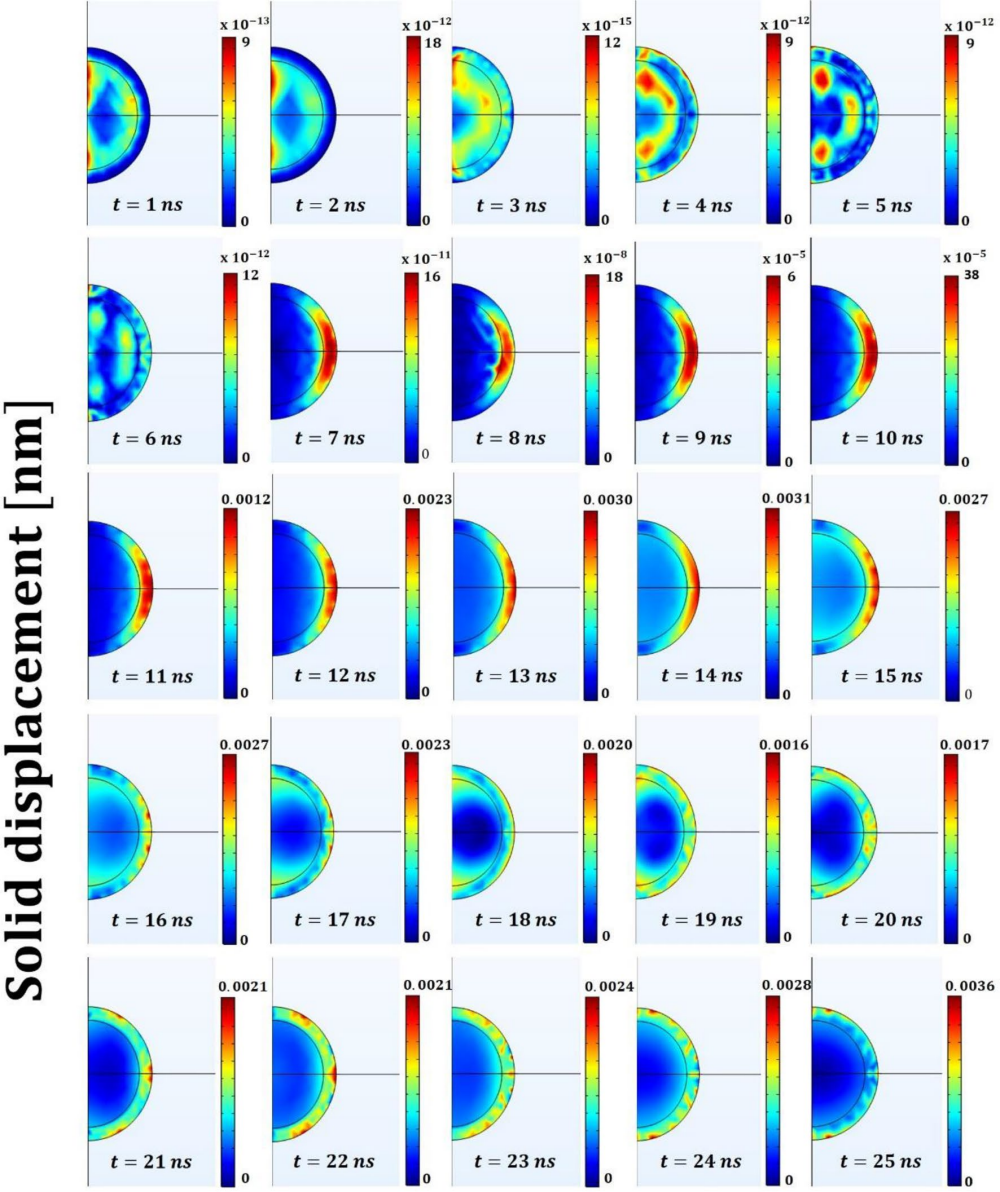
Solid displacement field within the polymeric (PDMS) core–shell micro-bead (which radius at rest is 500 [nm]) for the initial 25 ns of the process. Remarkable is the displacement of the bead’s shell in the equatorial region during the release of the strain energy. The colorbars have units of nanometers [nm].

In order to clarify the temporal dynamic of the RF pulse excitation and of its subsequent conversion into heat we find useful recalling several fundamental premises behind the concept of *RF-metamaterials*^1^. These *metamaterials* have been fabricated by adding carbon-based nanomaterials (or nanostructured materials) to polymeric or dielectric matrices in the aim of operating in the radiofrequency domain. In particular, the doping components are randomly dispersed in the host and, for *high concentration,* they allow overcoming the percolation threshold leading to a metallic-like conduction within the metamaterial. Specifically, these materials allow achieving negative permittivity and consequently a plasma-like behavior resembling that of metals in the optical regime. The possibility of obtaining plasma-like dispersion within these new metamaterials in the MHz range coupled with ultra-high field confinement $$\sim 0.5 {\lambda }_{opt}\times {10}^{-6}$$ (conventional plasmonics reaches $$\sim {\lambda }_{opt}\times {10}^{-2})$$ were theoretically proved in^1^. The result was an enhanced plasmon-excited resonant optical absorption $${\sigma }_{abs}$$ of the investigated micro-device and thus more intense mechanical vibration by frequency-overlapping tailored acoustic resonances. Based on this premises we can now analyse more in detail the heating process occurring upon the RF absorption by the micro-bead when illuminated by an incident pulse with Gaussian profile (see Eq. 4 and 5 in Sect. 2.2). We assumed a RF single pulse of (FWHM) 1.5 ns, fluence 40 J/m^2^ at 385 MHz (that gives an intensity of $$2.67e10 W/{m}^{2} )$$ and calculated the absorbed energy by Joule effect (i.e.$${Q}_{r}=\overrightarrow{J}\cdot \overrightarrow{E}$$) and the consequent temperature rise within the micro-bead (reported in Figs. 2 and 3). Since the temperature rise is due to the Joule effect, the conversion time of the electromagnetic (RF) pulse into heat depends on the dynamics of the plasmon and its interaction with the phonons^21^. This is expected to happen in at least one optical cycle (or possibly more) that is at least at 2.5 ns. We observe it in about 6–7 ns which is approximately 2–3 optical cycles (the optical forcing frequency is 385 MHz). In particular, from Fig. 2 (and Fig. 3) we observe that the heating process is highly confined in space: the temperature increase occurs prevalently in the shell region that embeds the CNT and falls to zero in less than 100 nm from the particle surface in the surrounding water^1^. This confirms that the plasma-like behaviour obtained with these new metamaterials in the RF-range allows achieving much higher optical absorption and consequently more intense mechanical vibration and stronger acoustic signals in the surrounding environment, than conventional photoacoustic. This becomes plastically evident in Figs. 4 and 5 for which the temperature difference illustrated in Fig. 2^29^ acts as the driving force of the wrinkling and the subsequent (quasi-periodic) snap-buckling phenomena, as discussed in detail in the following sections.

### Phase 1: Building of the inner thermal gradient that induces a wrinkling instability

For the system configuration with carbon nanotubes embedded in the shell (that is the heating element), the propagation of the heat waves generated by the pulsed laser, is primarily directed toward the core of the bead (being the path of minimum thermal resistance). Therefore, as illustrated in Fig. 2, after few nanoseconds a non-negligible temperature difference (of the order of several milli-Kelvin) is built between the shell and the core on one side, and the shell and the water on the other side. In addition, Fig. 3 shows that after few nanoseconds the most part of the thermal energy is already stored in the micro-bead’s core. Therefore, the micro-bead’s shell is in a mechanical state favorable to undergo a wrinkling instability. Wrinkling instabilities occurs in elastic membranes as the result of in-plane compression and their wavelength is fully determined by the geometric and elastic properties of the medium. Therefore, the tuning of these features allows achieving wrinkles’ wavelengths spanning the range from nanometers to many micrometers^15^. Exploiting these features, many recent works relied on controlled wrinkling for creating nano- and micro-scale patterned surfaces. In particular, their features of spatial periodicity, and the resulting wave interference phenomena, allow achieving unique optical and acoustic properties. Examples of wrinkling are common in nature to induce beneficial effects such as turbulent drag reduction^30^ or self-cleaning properties^31^. The occurrence of wrinkling instabilities is mathematically treatable in closed-form in case of thin film bonded onto compliant substrate^15,16^. In essence, when in-plane compression in an initially flat structure reaches certain critical values because of mechanical loading, thermal expansion mismatch or chemical reactions, wrinkling reduces the elastic strain energy in the film layer and lessens compressive strain^16^. The quasi-hexagonal wrinkling pattern reported in the present study is consistent with the results available in literature^32,33^ for core–shell spherical systems undergoing thermal wrinkling.

The plots reported in Figs. 4 and 5 (describing the acoustic pressure field and the solid displacement field, respectively) plastically show the buckling phenomenon and the emergence of a “close-to-hexagonal” tessellation pattern. On the other hand, when the core is the heating element, the resulting thermodynamic features of the system cannot overcome the critical conditions to create a wrinkling pattern^15,16,29^. This *wrinkling instability* is central to the overall phenomenon reported because it drives the creation of coherent structures (namely the tessellation) which, once disrupted from the nonlinear interactions, are regenerated by this very instability phenomenon^29^. As discussed in the introduction, the storage of thermal energy in the micro-bead’s core plays the role of “noise-source” in the observed bistable resonating mechanism. The amount of stored energy (i.e. its frequency content) should be such to induce a switch-rate frequency (i.e. the time scale of cell formation) matching that of the (acoustic) forcing frequency^29^.

A fundamental study to support this claim is provided by the work of Cao et al.^29^ In fact, most of the studies available in literature on the spontaneous formation of ordered buckling patterns in thin films bonded on compliant substrates focused on the buckling occurring in planar geometries, neglecting the effect of the substrate curvature. The authors of ^29^ highlight that the buckling behavior on a closed surface will differ from that on a surface with free boundaries, since they are topologically distinct geometrical objects (the first has genus zero while the second genus one). Therefore, the buckling patterns on a sphere will manifest some features primarily arising from the topological constraint. The authors investigated the possibility of controlling thin film buckling patterns by varying the substrate curvature and the stress induced therein upon cooling. The investigation reports numerical and experimental results based on a spherical Ag core*/*SiO_2_ shell system with total diameter few microns. In particular, they considered a spherical substrate of radius R, Young’s modulus ${E}_{s}$ and Poisson’s ratio ${\nu }_{s}$ on which is bonded a film of uniform thickness h, Young’s modulus $E$ and Poisson’s ratio $\nu$. The authors observed (both numerically and experimentally) that as the temperature is lowered by a magnitude $\Delta T$, the substrate contraction imposes a radial pressure on the interface. From the continuity of pressure and circumferential strain at the interface, the authors derive a closed-form expression for the magnitude of the compressive hoop stress in the film. The authors concluded that, once the ratio $\frac{R}{h}$ is assigned, the nominal stress ${\sigma }_{f}$ within the film will keep growing with increasing $\Delta T$ until the film undergoes buckling once a critical stress ${\sigma }_{c}$ is reached. The consequence is a partial relief of the hoop stress. Then, by further increasing $\Delta T$, the buckling amplitude grows and the buckling pattern modifies in the aim of minimizing the total strain energy. In particular, the authors obtained a map of the buckling patterns when varying the normalized substrate curvature vs the nominal film stress. We merely observe that the occurrence and the evolution of the buckling pattern reported in this study are fully compatible with the results reported in^29^. More importantly, the authors of^29^ observed that, especially for larger particles, the buckles formed immediately after the film being overstressed and persisted with the increasing film stress. Also in this study, the process of buckling formation restarts immediately after the (quasi-)periodic interruption due to the interaction with the acoustic standing waves that provides a (quasi-)periodic decrease of the hoop stress (via the collection and discharge of the strain energy toward the surrounding water).

We recall that the response of bistable systems showing stochastic resonance, shows remarkable periodicity only when the noise level is such to cooperatively concur with the periodic forcing in order to make almost exactly one switch per period (this correspond to a maximum in the signal-to-noise ratio). When the noise level is too low or too strong, i.e. when there are very few or too many switches per period of the forcing sinusoid, there is no periodicity in the system response.

### Phase 2: Interfering of the (acoustic) standing waves that set a snap-through-buckling mechanism

Concurrently with the previous phenomena, standing waves associated with the (laser-) activated acoustic resonant mode of the micro-bead, vibrate circumferentially along the bead. The result of their interference is the activation of the energy barrier of the buckled cells. This process is non-linear. The bistable resonating bead therefore reaches the “high-state” that corresponds to an organized disruption of the tessellation. The model and data presented in this work only account for thermo-elastic damping that is due to the stress inhomogeneity raised by the microscopic heat conduction but assume absence of structural damping. We then analyzed a real dissipative system by using the Rayleigh damping model with a loss coefficient $\eta =0.1$ ^38^, which is a realistic value for PDMS in this low-frequency regime. The generated pressure oscillations are reduced about 57% in magnitude and are completely damped down after 500 ns.

We recall that Rayleigh damping is a frequency-dependent damping model that can be applied in time-dependent simulations. It depends on two input parameters ${a}_{0}$ and ${a}_{1}$ that, once given the values of $\eta {\text{and}} \omega ,$ can be determined from the following equation: $\eta =\frac{1}{2}\left(\frac{{a}_{0}}{\omega }+ {a}_{1}\omega \right),$ where $\eta$ is the loss coefficient. Let us consider for example the case where the micro-transducer is working within a range of frequencies $[{\omega }_{1},{\omega }_{2}]$. To calculate the two parameters ${a}_{0} {\text{and}} {a}_{1}$ are then necessary two sets of $(\eta ,\omega$). In the present work we are working in the frequency band of few hundreds MHz that is a low frequency regime compared to common applications of this type of polymer (or similar) in the GHz range^34–37^. Therefore we enforced the same values of $\eta$= 0.1 ^38^ at the extremal values of the investigated frequency range ${(i.e. @ \omega }_{1} \mathrm{and }@{\omega }_{2})$ and this return the sought values of ${a}_{0} {\text{and}} {a}_{1}$.

In the case the core is the heating element (i.e. doping CNT are embedded solely in the core and the micro-bead does not have a plasmonic resonance) and the shell is an electric insulator, the switching mechanism is extremely weak.

We remark that the (hysteretic) succession of Phases 1 and 2 corresponds to a “snap-through-buckling” mechanism. The features of such mechanisms are well documented in literature. For example, several recent studies^33,39,40^ have analyzed theoretically and experimentally the conditions of occurrence of (snap-) buckling mechanisms in multi-layered polymeric spheres characterized by the "close-to-hexagonal" deformation pattern identified in this study.

The overall dynamic of this mechanism is plastically represented in Fig. 2, 4 and 5. The snapshots of these Figures, report respectively the temporal evolution of the acoustic pressure field, the temperature field and the solid displacement field for the initial 25 ns. In particular, during the initial time instants a snap-through-buckling mechanism already occurs (e.g. nanoseconds^2–7^ of Fig. 4). In this phase, the efficiency of the process is very low, with a maximum value of the generated pressure around 10^−6^ Pascal (mainly because of the low temperature gradients). However, this initial dynamic plays a crucial role in triggering the following stochastically resonant (thermo-acoustic) oscillations, during which the amplitude of the pressure oscillations rises to tens of Pascal. The pumping effect based on the “snap-through-buckling” mechanism in fact becomes dramatically evident after nanosecond 8 of Fig. 4.

### Phase 3: Releasing of the elastic deformation energy in the form of focused acoustic shot

Finally, the strain energy collected by the standing waves by disrupting the tessellation is released in the form of a focused acoustic shot. The disruption of the tessellation starts from the larger cells of the polar region (along the laser direction) and propagates until reaching the smaller cells of the equatorial region (orthogonal to the laser direction) where the maximum displacement occurs (shot-like). This mechanism causes the release of highly focused strain energy, as dramatically evident in the snapshots at nanoseconds 13 and 22 of Figs. 4 and 5. After the acoustic shot, the released strain energy radiates away in the surrounding liquid in the form of spherical pressure waves. Thus, the system recover the status to undergo Phase 1 and prepares for the next cycle.

### Dynamical characteristics of the phenomenon

**Figure 6:**
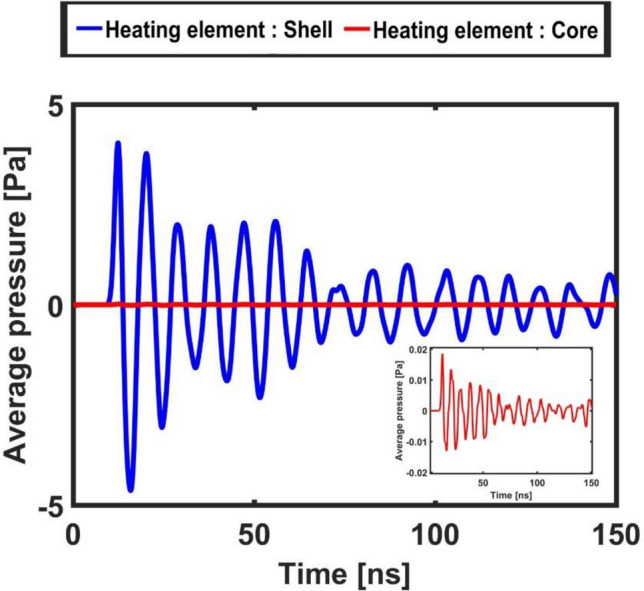
Average pressure measured on a spherical surface of radius 6 times the core–shell micro-bead. The blue curve refers to the case with shell as the heating element and the red curve refers to the case with the core as the heating element (enlarged in the inset).

**Figure 7:**
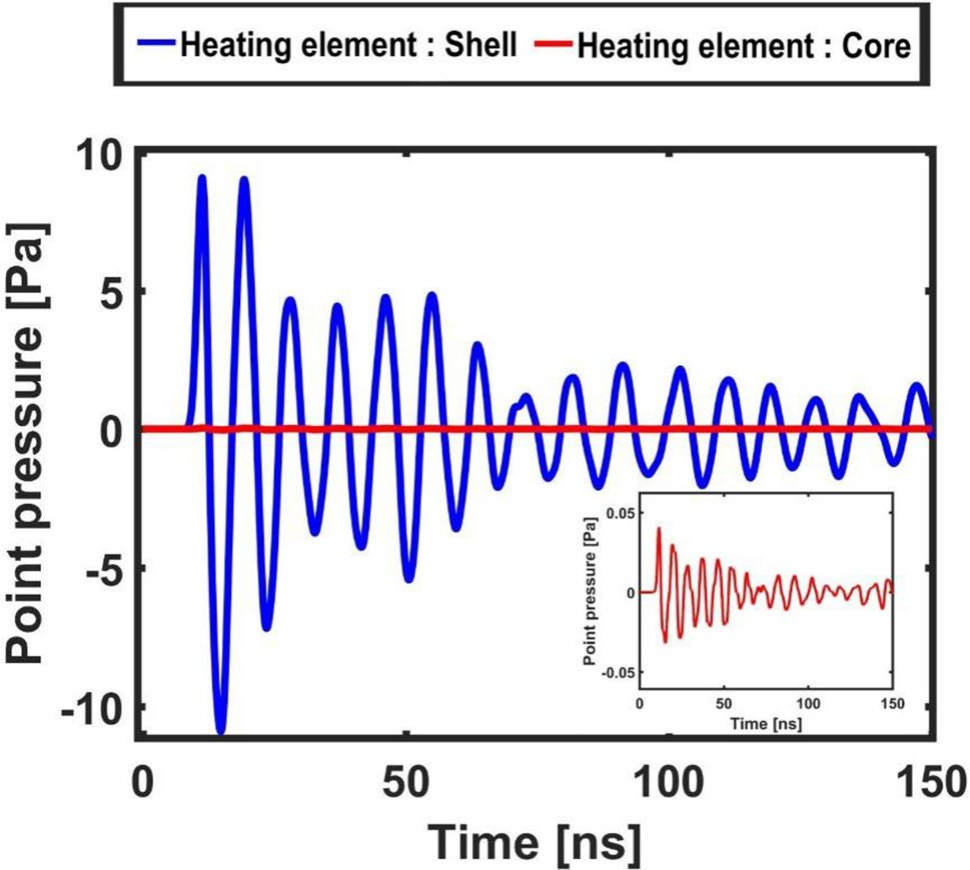
Point pressure value measured in the surrounding water environment at one bead’s diameter distance from its center (approximately the focus region of strain energy release). The blue curve refers to the case with the shell as the heating element and the red curve refers to the case with the core as the heating element (enlarged in the inset).

In this section are illustrated and commented the temporal evolution of the (surface-) averaged temperature, pressure, acceleration and displacement signals of the polymeric core–shell micro-bead in order to gain further insights in the observed phenomenon. Each dataset reports both the cases of 1) shell heating element and 2) core heating element (we recall that only in the first case the system shows a plasmonic resonance). Figures 6 and 7 report the values of the pressure oscillations averaged on a spherical surface of radius six times the micro-bead and at a point one bead radius away from its surface, respectively. The first case has been selected to illustrate how the average amplitude of the oscillations decays when propagating in water, while the second approximately coincides with the focus region of the strain energy release. Both Figures clearly remark the emergence of a non-linear (quasi-)periodic response of the system, that are distinctive features of bistability. The “higher-order (non-linear) behavior” of the pressure oscillations illustrated in these Figures is a distinctive marker of the intermittency that characterizes stochastically resonating bistable systems. In fact, as already discussed in the introduction, the concurrent effect of noise and small periodic perturbations concur to determine an order increase in the system response^41^. These plots show that pressure oscillations of amplitude as large as tens of Pascal can be generated by a polymeric micro-bead of diameter 1 um (or smaller). In the insets of the Figs. 6 and 7 are reported the pressure oscillations obtained when the core is the heating element, i.e. when in the micro-bead has not been excited a plasmonic mode. A two orders of magnitude difference is revealed in the amplitude of the pressure oscillations with respect to the previous case.

**Figure 8:**
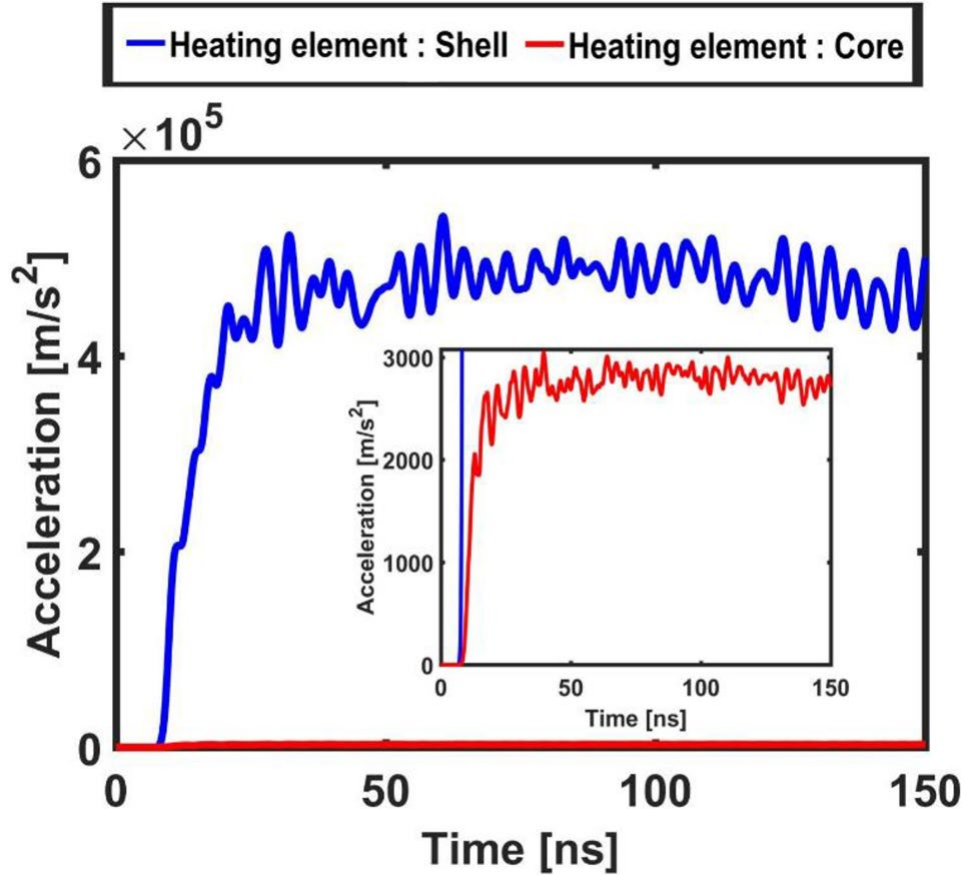
Acceleration of the core–shell micro-bead measured (averaged) on its surface for the case of shell heating element (blue curve) and of core heating element (red curve enlarged in the inset).

**Figure 9:**
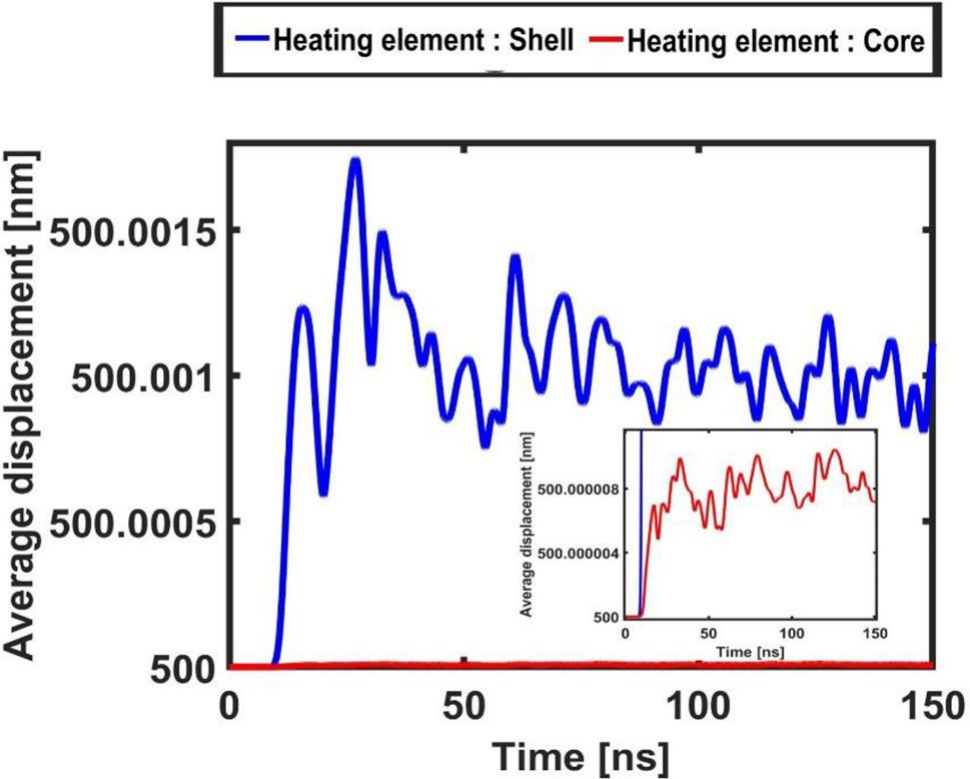
Displacement of the core–shell micro-bead (relative to its radius at rest of 500 [nm]) measured (averaged) on its surface for the case of shell heating element (blue curve) and of core heating element (red curve enlarged in the inset).

More information on the micro-bead’s dynamic can be gained looking at Figs. 8 and 9 that illustrate the temporal evolution of its acceleration and displacement, averaged on its surface, respectively. These plots dramatically illustrate a strongly intermittent and impulsive dynamic that, overall, shows remarkable characteristics of temporal (quasi-)periodicity.

Also for these Figs. 8 and 9, the insets report a zoom in on the signals calculated for the case of core heating element. A difference of two orders of magnitude in the amplitude of the signals is read with respect to the case when the heating element is the shell.

This behavior can be interpreted by looking at the system from a thermodynamic perspective, i.e. analyzing the temporal evolution of the maximum temperature values in its constitutive elements, i.e. the core, the shell and the overall bead. Because of the planar symmetry of the system with respect to the direction of the laser beam (see for example Figs. 2, 4 and 5) the maximum temperature values along a fixed radial direction (the horizontal-axis) were measured and compared. The maximum temperature values (reported as the difference with respect to the temperature of the surrounding water 293.15[K]) calculated on the inner core radius, on the outer shell radius and on the overall bead radius are reported in Fig. 3. As in the previous Figures, as a term of comparison, are reported (enlarged in the inset) the same distributions calculated in the case of the core heating element.

Figure 3 shows that, when the core is the heating element, i.e. the system does not have a plasmonic resonance, the maximum temperature occurs in the shell’s volume. Therefore most of the thermal energy generated by the laser pulse is not efficiently stored making the resulting thermo-acoustic oscillations of very modest intensity (as illustrated in the inset of the accelerogram in Fig. 8). On the other hand, when the shell is the heating element (i.e. in the system is excited a plasmonic resonance), the storing effect on the generated thermal energy is very efficient (as shown by the marked peaks in Fig. 3) and efficient is the resonant (thermo-acoustic) switching mechanism.

Figure 3 plastically shows that, when the shell is the heating element, after 78 ns the curve describing the micro-bead’s maximum temperature (red curve) coincides with that of the core (blue curve). Therefore, the thermal energy is stored very efficiently and the system shows an average temperature difference with respect to the surrounding water of several milli-kelvin for hundreds of nanoseconds. When instead the core is the heating element, the overall micro-bead’s maximum temperature coincides with that of the core only after 500 ns (not shown in Fig. 3), when most of the thermal energy has been already convected away to the surrounding environment. The maximum temperature of the system in this case is two orders of magnitude lower than in the previous case. The photoacoustic conversion efficiency, calculated as the ratio of the photoacoustic pulse energy over the laser pulse energy^42^, shows a difference of five order of magnitude for polymeric micro-beads where the described stochastic resonant phenomenon occurs (that is in presence of coupled optical-thermo-acoustic resonances) with respect to that where it does not.

We just recall that the key feature for activating the doubly resonant mechanism lies in the frequency (i.e. the time-scale) of the RF-excitation pulse. We showed that only at specific spectral positions (double matching the plasmonic and acoustic resonances) a RF pulse is capable of heat generation (and therefore of temperature gradients) within the micro-transducer sufficient for activating the quasi-periodic acoustic oscillations. Therefore, if the transducer is submerged in a larger environment characterized by temperature fluctuations of milli-Kelvin amplitude (thus comparable with those within the micro-transducer), the temporal and spatial scales of these fluctuations are decoupled by those occurring within the micro-transducer along the reported stochastic emission process. Therefore, we expect these temperature fluctuations to have no effect on the reported process.

**Figure 10:**
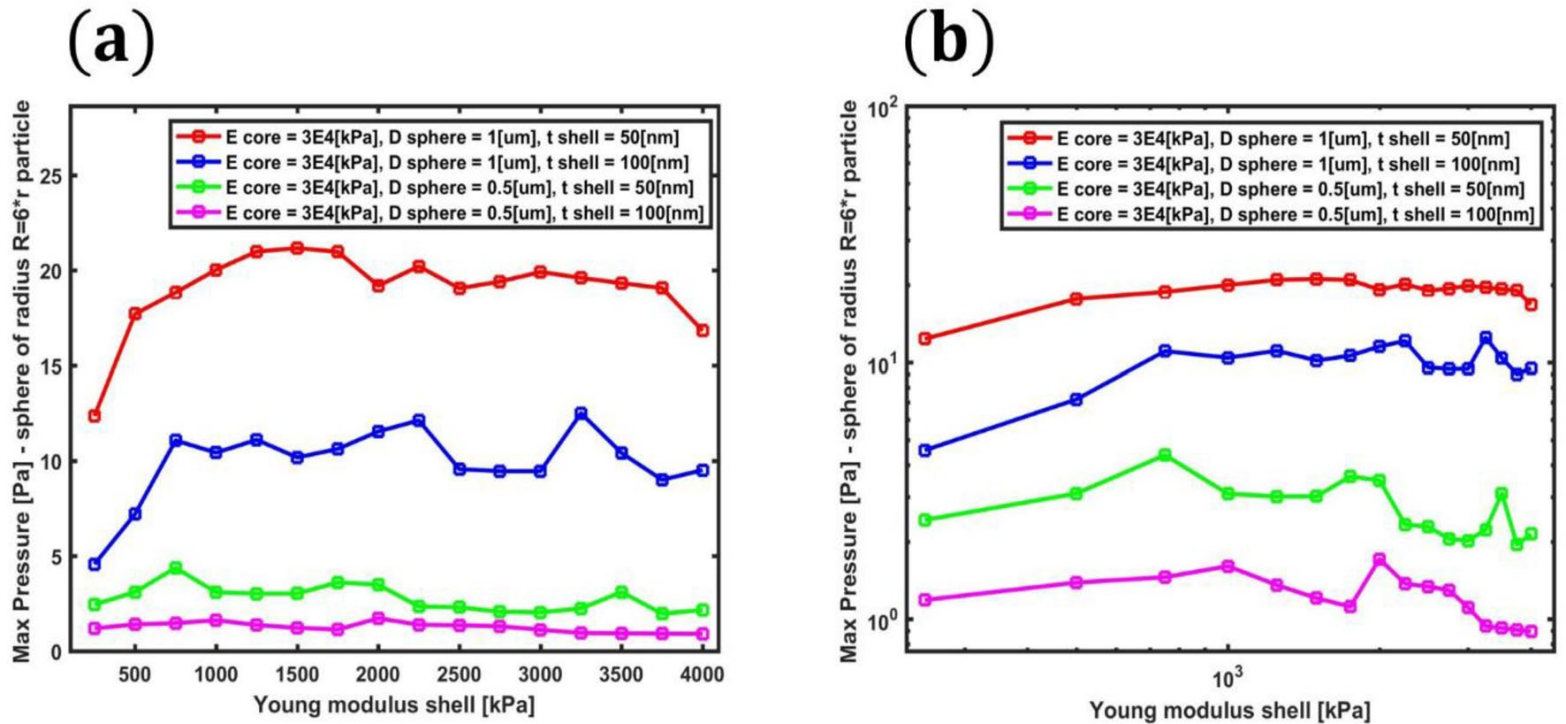
Parametric study showing the maximum pressure values generated when decreasing the bead’s diameter and the thickness of its shell versus the shell Young’s modulus. The Young’s modulus of the core is set to 3E4 kPa. Plots are in (**a**) linear and (**b**) logarithmic scales.

A parametric study was also performed in the aim of calculating the maximum pressures achievable when decreasing the micro-bead’s diameter and the thickness of its shell. The maximum pressure generated via the above-described stochastically resonating (optical-thermo-acoustic) bistable mechanism was calculated for a bead of total diameter 1 um with shell thickness of 100 nm and 50 nm. Then the total diameter was reduced to 0.5 um and the study repeated. Extrapolating the data of this study (reported in Fig. 10), it is possible to infer that polymeric beads with diameter below a quarter of a micrometer would produce pressures oscillations of few decimals of Pascal.

To make a comparison between the pressure generated by the proposed micro-transducer and other commonly used ultrasound contrast agent^43,44^ it is necessary evaluating the Photoacoustic signals obtained at the same distance. This comparison is typically made at mm-distances from the particle’s center^1^. As a term of comparison, we will use the results of Prost^45^, following the approach proposed in^1^. To obtain the possible measurements at 1 mm it is possible to use the conversion formula (valid for spherical waves)${P}_{{\text{max}}@ 1 mm}={P}_{max} \frac{1.1 R}{1 mm}$. We can make a rough comparison between the values reached in this study-case (largely optimizable as shown in Fig. 10) for an incident pulse with FWHM = 1.5 ns and fluence = 40 J/m^2 with those reported by Prost for a 40 nm-gold nanoparticle^45^ using a pulse with FWHM = 5 ns and fluence = 10 J/m^2. In the study-case analysed in the present work we obtained ${P}_{{\text{max}}@ 1 mm}=39 \left[Pa\right]\frac{1.1 R}{1 mm}=0.02145$ which is about 3 times the values reported in Prost^45^, suggesting that the efficiency of the proposed system, when using nano-second pulse excitation, is comparable (or higher after simple optimizations) with the plasmonic nanoparticles one. In addition, to enhance the contrast in imaging, it is possible to envision a system of nearby micro-beads.

We remark that this seminal work is part of a broader investigation aimed to designing and fabricating optical contrast agents of nanometric size. In this respect, we recall that the reported numerical study has to cope with more than six orders of magnitude difference between the exciting radio-wavelength and the characteristic size of the optical transducer investigated. Consequently, for particles of radius below 250 nm and shell thickness below 50 nm we had severe convergence issues with all the applied Finite Elements solvers.

In summary, the approach proposed in this study introduces a new paradigm for light-to-sound conversion in extreme subwavelength scales. Notably, it can be exploited for concrete applications in disparate fields. For example, in medicine it can be exploited for the development of radically new systems for ultra-low-invasive and sensitive diagnosis of diseases, and in developing new molecular designs for advanced theragnostics. In toxicology and nanosafety can be used for better understanding the behavior of nanostructured metamaterials alone and in combination with other functional biomolecules loaded into the polymer matrix. In biophysics and physiology can be applied for reaching a never-tested RF penetration depth into tissues, thus promoting better knowledge on light-biomatter interactions in-vivo. More generally, the proposed approach has the potential to develop a complete and entirely novel platform for accurate diagnosis and specific therapy in a unique and integrated system that provides super-resolution microscopy technique seeing the world in RF never seen before. We foresee important applications also in neuroscience in which the delivery of light deep into the brain remains a fundamental challenge while RF is reported to interact directly with neurons in deep brain. We cannot ignore that the new RF transduction concepts suggest also immediate possibilities of new sensing techniques for telecommunication and radar applications.

The concrete applicability of the proposed micro-transducer in the bio-related fields listed above can only take place only after its biocompatibility verification. For what concerns the present study the biocompatibility considerations mainly boil down to the biocompatibility of the carbon nanotubes (CNT) embedded in its shell. We recall that here we mainly focused on the concept of the conversion of EM (radio-) pulse into pressure, which does not depend on the presence of the nanotube itself but on the plasmonic response of the system. This can be generated with CNT but in principle, also with other materials. A comprehensive review on the available (and often conflicting) investigations into the cytotoxicity and biocompatibility of CNT is provided in^46^. The authors of^46^ summarize the results of numerous published studies into CNT-based biomaterials, which support the biocompatibility of CNT and CNT-based materials. These studies mainly focused on the interactions between CNT-based materials, neural cells, osteoblasts, fibroblasts, antibodies and the immune system, ion channels and cellular membranes. However, the authors concluded that still much work needs to be done in establishing the toxicity and biocompatibility of CNT. In the aim of minimizing these biocompatibility issues, we chose thermo-polymers (or similar responsive materials) as the matrix material for the proposed contrast agent. This choice is dictated by the fact that thermo-polymers dissolve at low temperature (45–50 °C). Their dissolution can be temporally and spatially controlled upon photo-induced stimulation of the micro-bead. Moreover, their dissolution can be locally monitored through the modification of the plasmonic (optical) signal. Importantly, the polymer dissolution causes the breakdown of the whole nanostructure, which will assure the systemic clearance of the micro material by the renal function.

## Conclusions

In this study was discussed the occurrence of a stochastically resonating (optical-thermo-acoustic) bistable mechanism in polymeric (PDMS) micro-beads size with stiff core and soft shell. The system is excited with a pulsed laser which characteristics are set to match both an optical and acoustic resonance of the bead (namely 385 MHz). The heat generated via the laser interaction with carbon nanotubes embedded in the bead’s shell, builds an inner thermal gradient that sets the conditions for the occurrence of a wrinkling instability. This instability drives the creation of an organized pattern in the form of a quasi-hexagonal tessellation. On synchronous time scales, the standing waves associated with the (laser-) activated acoustic resonant mode provide the flipping spike necessary for the organized disruption of the buckled cells forming the tessellation. The strain energy of these very cells, once collected by the standing waves is released in the form of a focused acoustic shot. Overall, the synergetic interplay of these phenomena corresponds to a snap-through-buckling mechanism that produces pressure waves characterized by high intensity and remarkable quasi-periodicity. The approach developed in this study introduces a new paradigm for light-to-sound conversion in extreme subwavelength scales exploitable for applications in the most disparate fields ranging from medicine to toxicology and nanosafety, from biophysics and physiology to the neuroscience.

## Data Availability

The data that support the findings of this study are available from the corresponding author upon reasonable request.
